# Flavin-Dependent Redox Transfers by the Two-Component Diketocamphane Monooxygenases of Camphor-Grown *Pseudomonas putida* NCIMB 10007

**DOI:** 10.3390/microorganisms4040038

**Published:** 2016-10-13

**Authors:** Andrew Willetts, David Kelly

**Affiliations:** 1 College of Life and Environmental Sciences, University of Exeter, Exeter EX4 4QG, UK; 2 School of Chemistry, Cardiff University, Cardiff CF10 3AT, UK; D.J.Kelly@cardiff.ac.uk

**Keywords:** diketocamphane monooxygenase, flavin reductase, putidaredoxin reductase, *Pseudomonas putida* NCIMB 10007

## Abstract

The progressive titres of key monooxygenases and their requisite native donors of reducing power were used to assess the relative contribution of various camphor plasmid (CAM plasmid)- and chromosome-coded activities to biodegradation of (*rac*)-camphor at successive stages throughout growth of *Pseudomonas putida* NCIMB 10007 on the bicylic monoterpenoid. A number of different flavin reductases (FRs) have the potential to supply reduced flavin mononucleotide to both 2,5- and 3,6-diketocamphane monooxygenase, the key isoenzymic two-component monooxygenases that delineate respectively the (+)- and (−)-camphor branches of the convergent degradation pathway. Two different constitutive chromosome-coded ferric reductases able to act as FRs can serve such as role throughout all stages of camphor-dependent growth, whereas Fred, a chromosome-coded inducible FR can only play a potentially significant role in the relatively late stages. Putidaredoxin reductase, an inducible CAM plasmid-coded flavoprotein that serves an established role as a redox intermediate for plasmid-coded cytochrome P450 monooxygenase also has the potential to serve as an important FR for both diketocamphane monooxygenases (DKCMOs) throughout most stages of camphor-dependent growth.

## 1. Introduction

**Figure 1:**
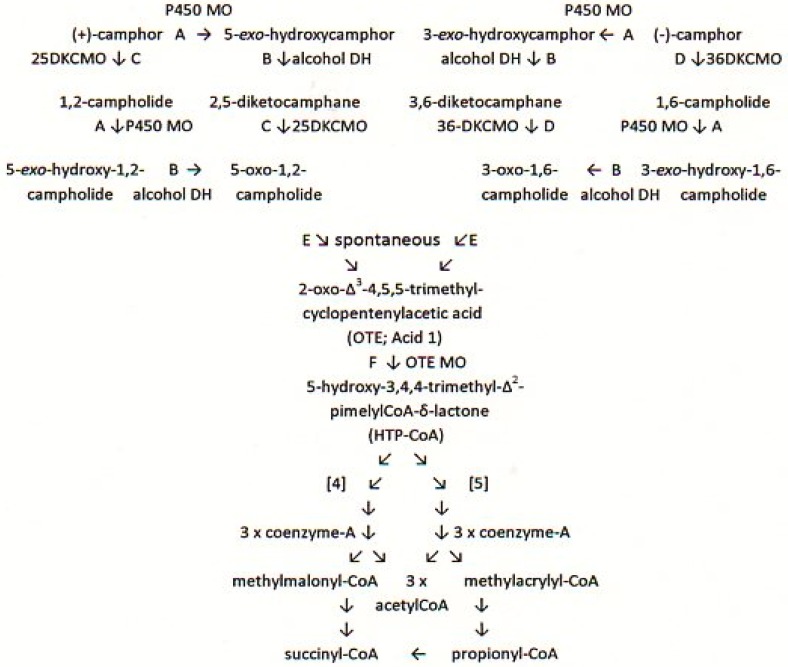
Pathway(s) of (+)- and (−)-camphor degradation in *P. putida* NCIMB 10007. A = cytochrome P450 monooxygenase (*camA* + *camB* + *camC*); B = *exo*-hydroxycamphor dehydrogenase (*camD*); C = 2,5-diketocamphane 1,2-monooxygenase (*camE_25-1_* + *camE_25-2_*); D = 3,6-diketocamphane 1,6-monooxygenase (*camE_36_*); E = spontaneous; F = OTE monooxygenase (*camG*). The early steps preceding the formation of OTE represent suggested possible alternative combinations of the same enzymes A–D; the late stages beyond HTO-CoA remain incompletely characterised, with two possible alternative catabolic sequences suggested [4,5] to account for the assimilation of the 10 carbon atoms of the bicyclic monoterpenoid into the TCA cycle as 1 × C4 succinyl-CoA plus 3 × C2 acetyl-CoA esters.

The bicyclic monoterpenoid camphor, present as either separate enantiomer or a racemic mixture, is a molecule that is encountered relatively rarely in the biosphere [1]. It was for this reason that camphor was chosen to initiate a series of studies on microbial biodegradation as a model of how an “atypical” molecule can become integrated into the TCA cycle and other shared central pathways of metabolism [2,3]. Although investigation of camphor metabolism by one specific microorganism, *Pseudomonas putida* NCIMB 10007 (= C1B, = PpG1, = ATCC 17453) has continued for almost 60 years, resulting in more than 50 publications on the subject, important details of some of the relevant enzymes whereby camphor is oxidised to central pathway intermediates are still unclear (Figure 1).

Establishing the contribution of the known enzymes to the overall pathway poses some potentially challenging problems. Firstly, some [4], but importantly not all [5], of the relevant genes are carried on the large (533 kbp) linear CAM plasmid. Secondly, physiological and genetic evidence has shown conclusively that a number of the key enzymes are subject to elements of transcriptional control at different times throughout the successive phases of growth of *P. putida* NCIMB 10007 on camphor-based defined media [6,7]. It is unfortunate that despite these known characteristics of *P. putida* NCIMB 10007, biochemical studies have focused almost exclusively on enzymes present in cells harvested in the very late phase of logarithmic growth immediately prior to entering into the stationary growth phase, and have failed to distinguish between the plasmid- and chromosomal-coded origin of the relevant activities. A key equivalent step in the early part of the separate degradation pathways for each camphor antipode is the ring expansion of the relevant chiral diketocamphanes to the corresponding unstable lactones by isoenzymic diketocamphane monooxygenases (DKCMOs). Subsequent spontaneous hydrolysis of the chiral lactones serves in each case to open one of the two fused alicyclic rings of the bornane carbo-skeleton, and thereby converge the two branches of the camphor degradation pathway by forming the common achiral hydroxy acid 2-oxo-Δ^3^-4,5,5-trimethylcyclopentenylacetic acid (OTE).

Although previously reported to be flavoproteins containing bound flavin monooxygenase (FMN) and classified along with a relatively few other similar enzymes as so-called Type II Baeyer-Villiger monooxygenases [8], a recent study [5] has demonstrated conclusively that in late log-phase cells of camphor-grown *P. putida* NCIMB 10007 both 2,5- and 3,6-DKCMOs operate as members of a wider class of oxygen-dependent enzymes, the two-component monooxygenases (TCMOs; [9]). Most significantly, the relevant plasmid-coded homodimeric oxygenating moieties for both DKCMOs receive unbound reduced flavin mononucleotide (FNR) by rapid free diffusion, not as previously believed from a plasmid-coded oxidase/dehydrogenase [10,11], but from Fred, a chromosome-coded enzyme [5]. Fred is not a flavoprotein but a homodimeric reduced nicotinamide adenine dinucleotide (NADH)-dependent flavin reductase (FR) for which FMN serves as a substrate rather than a coenzyme, as shown for other microbial FRs [12]. As well as finding no evidence for a relevant CAM plasmid-coded reductase or oxidase/dehydrogenase, Iwaki et al., also showed that the CAM plasmid contains two genes, *camE_25-1_* and *camE_25-2_*, with very similar sequences that code for isoenzymic versions of 2,5-DKCMO that almost certainly arose from gene duplication and subsequent divergence [5]. The extremely high purity (>99.9% ee) of the chiral products of biotransfomations catalysed by a mixture of the two 2,5-DKCMO isoenzymes [8,13,14] strongly suggests that both enzymes have very similar catalytic specificities.

Taking the ability to promote NADH-dependent biooxidation of ketones to lactones by highly purified preparations of DKCMOs as the relevant parameter, it was subsequently further confirmed [15] that native FNR-generating enzymes other than Fred could serve such a role for the DKCMOs with equal or even greater efficiency during growth of *P. putida* NCIMB 10007 on camphor-based defined media. The principal relevant activities were shown to be two constitutive ferric reductases, chromosome-coded enzymes which have been shown previously to serve as FRs able to generate freely diffusible FNR in *P. putida* KT2440 [16,17]. Somewhat surprisingly, putidaredoxin reductase (PdR) was also found to serve effectively in such a role. PdR is the plasmid-coded NADH-dependent flavin adenine dinucleotide (FAD)-containing flavoprotein that in cooperation with putidaredoxin (Pdx) serves a well-established role [18,19,20] as the sole supplier of reducing power to camphor *exo*-hydroxylase, the CAM plasmid-coded initial monooxygenase of the camphor degradation pathway. The equivalent deployment of alternative FRs able to generate freely diffusible FNR for subsequent use by a particular monooxygenase moiety has been recorded previously with other TCMOs, including various bacterial luciferases [21], nitrilotriacetate monooxygenase from *Chelatobacter heintzii* [22], dibenzothiophene-desulfurizing monooxygenase from *Rhodococcus erythropolis* [23], and some of the “tailoring enzymes” involved in the late stage synthesis of the polyketide antibiotics pristinamycin by *Streptomyces pristinaespiralis* [24], valanimycin by *Streptomyces viridifaciens* [25], and actinorhodin by *Streptomyces coelicolor* [26].

The purpose of the present paper is to characterise more fully aspects of both the enzymology and regulation of FNR generation and deployment by relevant CAM plasmid-coded and chromosomal DNA-coded enzymes that contribute to diketocamphane catabolism at the various stages of camphor-dependent growth by *P. putida* NCIMB 10007. Collectively, the various outcomes clarify over 50 years of research by defining the potential contributions that various different monooxygenase-FR combinations native to camphor-grown *P. putida* NCIMB 10007 make to the biochemistry of bicyclic monoterpenoid catabolism.

## 2. Materials and Methods

### 2.1. Bacterial Strains, Culture Maintenance, and Growth Conditions

*P. putida* NCIMB 10007 (ATCC 17453) was maintained on a basal salts medium supplemented with 17.5 mM (*rac*)-camphor as the principal carbon source as fully detailed previously [27]. Diauxic growth was achieved by culture on an equivalent medium supplemented with 2.5 mM sodium succinate plus 15.0 mM (*rac*)-camphor [7], and the levels of the two substrates monitored respectively by reverse-phase HPLC (Agilent 1200, Agilent Technologies, Santa Clara, CA, USA) on a Primesep 100 column [28] and GC (Shimadzu GC-14A, Shimadzu Europe, Druisberg, Germany) on a 10% Carbowax 20 M column [14].

### 2.2. Purification of PdR, Pdx, 2,5- , and 3,6-DKCMO

Samples of highly purified PdR, and the biooxygenating subunits of both enantiospecific DKCMO isoenzymes were prepared at 4 °C using a BioLogic FPLC system (BioLogic10, Bio-Rad, Hercules, CA, USA) as fully described previously [14]. Pdx was recovered as a by-product of the same procedure by deploying the comprehensively reported method of Gunsalus and Wagner [29].

### 2.3. Purification of FMN-Reductase Activities

Samples of highly purified Fred were prepared at 4 °C using an LKB Biologic FPLC system deploying the three-stage protocol fully described by Willetts and Kelly [15]. A combined Frp1 plus Frp2 preparation was a by-product of the procedure, and this was resolved into separate highly purified Frp1 and Frp2 activities using the purification protocol developed and fully described by Halle and Meyer [30].

### 2.4. Kinetic Studies of Frp1, Frp2, Fred and PdR 

Enzyme activities were routinely assayed spectrophotometrically (Hewlett-Packard model 8452A, Hewlett-Packard, Palo Alto, CA, USA) under anaerobic conditions by measuring the initial rate of the decrease in absorbance at 340 nm as described previously [15]. For Frp1, Frp2 and Fred, each respective 3.0 mL reaction mixture containing 60 mM Tris/HCl buffer (pH 7.6), 10–100 μM NADH, and 2–30 μM FMN was placed in a cuvette (3.5 mL, 1 cm light path) equipped with a magnetic stirrer bar and rubber septum. Prepurified argon was introduced into the solution by means of a needle through the septum, and a second inserted needle was connected to a tube venting into mineral oil. After flushing the reaction mixture for 4 min to remove oxygen, 40–60 mU of the appropriate purified concentrated (ultrafiltration) argon-scrubbed enzyme was injected into the cuvette to initiate the enzymatic reaction. Kinetic parameters *K_m_* and *K_cat_* were determined by using a Lineweaver-Burk plot of the Michaelis-Menton equation under steady-state conditions. In equivalent assays for PdR, FMN was replaced either with cytochrome c (30 μM) plus Pdx (10 μM) or dichlorophenolindophenol (DCPIP) (30 μM). A molecular masses of 11.5 kDa was used to calculate the molar concentration of Pdx.

Equivalent assays were undertaken aerobically in reaction mixtures flushed for 4 min with air instead of argon prior to the addition of an aliquot of the enzyme to be tested. In experiments to establish any effect of excess FMN when assayed aerobically, the range of added flavin was expanded to 5–80 μM.

### 2.5. Reaction Mechanism of Frp1, Frp2 and Fred

The dependence of the NADH-dependent reactions catalysed by each enzyme on the concentration of both FMN and NADH when assayed anaerobically was investigated under steady-state conditions using a variation of the previously described assay used to perform kinetic studies. The sequential reaction mechanism of each bisubstrate-biproduct enzyme was confirmed by the nature of the intersecting pattern of the double reciprocal plots of initial velocities versus NADH concentration (16–100 μM) at various different fixed concentration (3–16 μM) of FMN [31].

### 2.6. Flavin Transfer by PdR

All procedures were performed anaerobically using argon-scrubbed solutions in a sealed stirred 3.5 mL cuvette adapted for continuous argon flushing and placed in a Jacomex glove box itself flushed with nitrogen treated to remove any traces of residual oxygen [32]. After prior flushing a 3.0 mL reaction mixture containing 60 mM Tris/HCl buffer (pH 7.6) and 50 mU PdR for 4 min, a 0.5 mL sample was removed and its absorption spectrum (300–560 nm) recorded. A 0.5 mL aliquot containing 8 mU PdR and 25 μM NADH was added to the reaction mixture, which was then flushed for a further 4 min prior to removing a second 0.5 mL sample. This sample was separated into <10 kDa and >10 kDa fractions by ultrafiltration using a 10 kDa membrane, and an absorption spectrum (300–560 nm) recorded for each separated fraction. The reaction mixture was then supplemented with a second 0.5 mL aliquot containing 25 μM FMN. After flushing and stirring for 4 min, a sample was removed, separated into <10 kDa and >10 kDa fractions by ultrafiltration, and an absorption spectrum (300–560 nm) recorded for each separated fraction.

A modification of the procedure was undertaken with putidaredoxin-reduced flavin adenine dinucleotide complex (PdR-FADH_2_) generated by photoreduction in the presence of EDTA under anaerobic conditions by the method of Massey and Palmer [33].

### 2.7. Flavin Binding to the DKCMOs and K_d_ Values for FMN and FNR

Spectrophotometric titrations of separate highly purified preparations of each DKCMO isoenzyme with FMN were conducted aerobically in a 3.5 mL cuvette (1 cm light path). To aliquots of enzyme (0, 12, 36, 72 and 96 μM) in 3.0 mL Tris-HCl buffer pH 7.6 was added 10 μM FMN and the absorption spectra (300–560 nm) recorded after 3 min. In each case the fractional absorbance changes at 440 nm were used to calculate the concentrations of both bound FMN and free DKCMO, and hence a relevant *Kd* value as fully described previously by Valton et al. [34]. Molecular masses of 81.3 kDa and 84.6 kDa were used to calculate molar concentrations of the 2,5- and 3,6-DKCMO isoenzymes respectively.

Spectrofluorimetric titrations (Jasco model FP6500, Jasco, Easton, NH, USA) of separate highly purified preparations of each DKCMO isoenzyme with FNR were conducted anaerobically in a 3.5 mL cuvette (1 cm light path). The excitation wavelength was set at 295 nm. Each respective 3.0 mL reaction mixture containing 60 mM Tris/HCl buffer (pH 7.6) and 10 μM enzyme was purged with argon for 4 min to remove oxygen prior to the injection of an aliquot (0–10 μM) of an FNR stock solution prepared anaerobically from FMN by EDTA-dependent photoreduction [33]. The resultant fluorescence spectra (310–410 nm) were recorded, and the corrected fluorescence intensity at 339 nm used to determine the *K_d_* value for FNR as fully described previously [34].

### 2.8. Biocatalytic Reactions with Combinations of Purified Enzymes

Biotransformations with the various combinations of purified enzymes shown in Table 1 were carried out in reaction mixtures (1 mL) containing Tris/HCl buffer (60 mM, pH 7.6), 0.1 mM NADH, 0.02 mM FMN, 30 mU formic dehydrogenase, 50 mM sodium formate and 1 mM of a relevant chiral substrate. In each case 0.5 mg of the relevant pure DKCMO was used and 40–60 mU of the purified concentrated (ultrafiltration) candidate reductase. The amount of substrate remaining after 180 and 360 min at 25 °C was determined by GC as fully detailed previously [34].

### 2.9. Reproducibility

All described procedures were repeated a minimum of 5 times unless stated otherwise with the same or equivalent cultures and purified enzyme preparations. A high degree of consistency was recorded both between replicate cultures and replicate measurements; results are therefore presented as averaged values.

### 2.10. Chemicals and General Procedures

Unless otherwise stated, all chemicals, enzymes, and reagents were purchased from Sigma-Aldrich (St Loius, MO, USA) or Thermo Fisher Scientific and (Loughborough, UK) were used without further purification.

## 3. Results

### 3.1. Specific Activities of Key Enzymes Involved in FNR Generation and Deployment throughout Growth of P. putida NCIMB 10007 on Camphor

Gunsalus et al. [6] established that when *P. putida* NCIMB 10007 was inoculated into a defined medium containing both succinate and (*rac*)-camphor-U-C14, diauxic growth was observed whereby all available succinate was completely utilised during the first growth phase, with no CO_2_ derived from camphor released before commencement of the subsequent phase of exclusively camphor-dependent growth.

**Figure 2:**
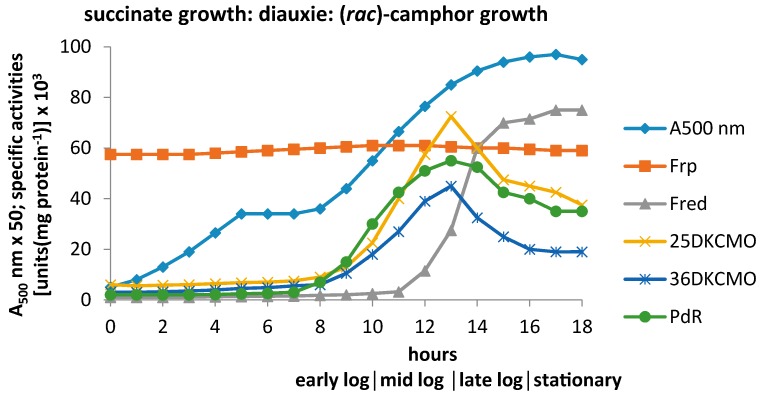
Changes in the optical density (A_500_ nm) and the specific activity of key enzymes of (*rac*)-camphor degradation during diauxic growth of *P. putida* NCIMB 10007 on a succinate plus camphor-based defined medium.

By extending these prior studies to monitor the specific activities of relevant enzymes in cell-free extracts prepared at timed intervals throughout these successive stages of growth, it should be possible to establish the importance of potentially key enzymes involved in FNR generation and deployment. As a preliminary step, each cell-free extract was subject to ultrafiltration using a membrane with a molecular weight cut-off of 40 kDa, and native polyacrylamide gel electrophoresis (PAGE) gels used to confirm the efficacy of each separation. Assays then conducted on both the resultant high and low molecular weight (MW) fractions. Averaged data from five replicate experiments conducted with a defined medium containing 2.5 mM succinate and 15 mM (*rac*)-camphor confirmed succinate depletion and the initiation of diauxic growth as coincident events (Supplementary Figure S1), and demonstrated that subsequent terpenoid-dependent growth promoted the induction of a number of established early enzymes of the camphor degradation pathway (Figure 2).

Of the FNR-generating enzymes assayed, the only constitutive activity detected was NADH-dependent ferric reductase (Frp), a result consistent with the a priori need for a means of generating intracellular ferrous ions from environmentally sourced ferric ions thereby contributing to successful aerobic growth [35]. Further studies (vide supra) confirmed that the activity consisted of two closely related monomeric Frps with MWs of 27.0 and 28.5 kDa, each of which could serve as an efficient FNR-generating enzyme for both DKCMOs. However, like the equivalent enzymes from *P. putida* KT2440 [36], they exhibited significantly different reduced nicotinamide coenzyme specificities.

By way of contrast, assays conducted on two other FNR-generating activities clearly indicated that they were both inducible enzymes present at very low levels in the initial succinate-dependent phase of growth, and thereafter subject to very different time-dependent profiles of induced activity during growth at the expense of camphor. One of these activities, located exclusively in the high MW fractions as confirmed by relevant native PAGE gels, promoted putidaredoxin-linked reduction of electron acceptors at the expense of NADH, and was induced in parallel with camphor *exo*-hydroxylase (Supplementary Figure S1). The principal protein present in the relevant samples was isolated (~51.0 kDa), analysed, and thereby confirmed to be identical to PdR [15], the monomeric protein product of the *camA* gene with a calculated molecular mass of 48.5 kDa [37,38]. The other inducible FNR-generating activity was located exclusively in relevant low MW fractions, and was only detectible in significant amounts from around the mid-log phase of camphor-dependent growth. Using previously described protocols [15], purification and subsequent characterisation confirmed that the principal protein in relevant low MW fractions corresponded to Fred, the chromosomally-coded 36 kDa NADH-dependent FNR-generating homodimeric enzyme isolated previously from camphor-grown *P. putida* NCIMB 10007 [5].

The principal FNR-dependent activities, the 2,5- and 3,6-DKCMO isoenzymes coded for by the *camE_25-1_* , *camE_25-2_*, and *cam E_36_* genes, were located exclusively in the high MW fractions. They were both clearly inducible activities present at levels sufficiently low to be within experimental error during the initial succinate-dependent phase of growth, but thereafter induced by camphor on a very similar time-scale to PdR, an active component of camphor *exo-*hydroxylase, the preceding monooxygenase in the camphor degradation pathway (Figure 1). It was apparent from the coincident levels of the various FNR-generating and FNR-dependent assayed activities that the oxygenating subunits of both isoenzymic DKCMOs could potentially form alternative active TCMO complexes with various candidate FNR-generating activities at different times throughout camphor-dependent growth.

Interestingly, an almost identical pattern of diauxic growth plus corresponding constitutive and inducible enzyme profiles was recorded when *P. putida* NCIMB 10007 was inoculated into a minimal medium containing succinate and 2-oxo-Δ^3^-4,5,5-trimethylcyclopentenylacetic acid (Supplementary Figure S2), which can be extracted in significant amounts from spent camphor-based media.

### 3.2. Relevant Kinetic Data for the Alternative FNR-Generating Activities in Camphor-Grown P. putida NCIMB 10007

#### 3.2.1. Constitutive Low MW FNR-Generating Activity: Ferric Reductases

By deploying the purification protocol developed for the Frps from various *Pseudomonas* spp. by Halle and Meyer [30], it was possible to prepare and subsequently characterise highly purified preparation of the 27.0 and 28.5 kDa Frps.

**Figure 3:**
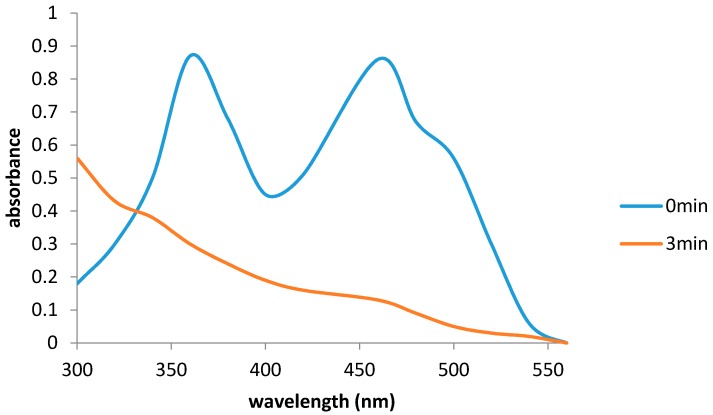
Frp1-dependent reduction of flavin monooxygenase (FMN) (40.0 μM) by nicotinamide adenine dinucleotide (NADH) (100.0 μM).

Initial kinetic studies undertaken under anaerobic conditions with NADH or NADPH as the electron donor, FMN or FAD or riboflavin as the electron mediator and DCPIP as the terminal acceptor confirmed that the 27.0 kDa enzyme (Frp1) can only effectively transfer reducing power from NADH (*K_m_* = 23 µM) to FMN, while FAD exhibited much lower activity and riboflavin was totally inactive. The *K_m_* (FMN = 6.9 µM, FAD = 68.8 µM) and *k_cat_* (FMN = 310 s^−1^; FAD = 996 s^−1^) values for the various active flavins suggested that the FMN + NADH combination was a comparatively effective means of generating FNR in camphor-grown *P. putida* NCIMB 10007. No significant activity was recorded by combining NADPH with any of the tested flavin electron mediators. Subsequent replacement of DCPIP with either ferric citrate or Fe^3+^-EDTA confirmed the additional role of Frp1 as a ferric reductase equally effective with both FeIII substrates. The fact that FMN was a substrate rather than a cofactor for the Frp1 was indicated by the complete reduction of an excess of FMN (25.0 µM) by NADH (100.0 µM) after addition of the enzyme (~2.5 U) under anaerobic conditions: the yellow colour of the reaction mixture disappeared and the relevant spectral change after 3 min indicated no residual FMN (Figure 3).

Equivalent studies undertaken with the 28.5 kDa enzyme Frp2 confirmed that this enzyme, like Frp1, can serve a dual role as an FR and an Frp for which FMN was the only effective electron mediator (*K_m_* = 8.6 µM; *k_cat_* = 358 s^−1^) when transferring reducing power from NADH. However, it was noticeable that with Frp2 both reduced nicotine adenine nucleotides could serve as electron donors, although the relative *K_m_* values suggested NADH (*K_m_* = 36 µM) would be a more effective reductant than NADPH (*K_m_* = 92 µM) in vivo. Spectral data almost identical to Figure 3 confirmed that Frp2, like Frp1, deployed FMN as a substrate rather than a cofactor.

Double reciprocal plots of the kinetic data generated by each purified Frp as a function of NADH concentration in the presence of various fixed concentrations of FMN (Supplementary Figure S3a,b) demonstrated that both enzymes exhibited similar kinetics: in each case, the intersecting lines at a point to the left of the vertical axis indicated that Frp1 and Frp2 were reductases that generated FNR by a sequential reaction mechanism [39]. These kinetic data for the Frps from *P. putida* NCIMB 10007 resembled that of ActVB, the FR component of the TCMO oxidoreductase that participates in the final step of actinorhodin synthesis in *Streptomyces coelicolor* [31].

When assayed under aerobic conditions, both Frps exhibited a marked progressive inhibition with added FMN concentrations higher than 20–25 µM (Supplementary Figure S3c), a phenomenon attributed to the effect on the enzymes of hydrogen peroxide generated abiotically from excess FNR by molecular oxygen. Corresponding progressive levels of peroxide production were confirmed in relevant stopped reaction mixtures using a pyrogallol and peroxidase-based test kit [25].

#### 3.2.2. Inducible Low MW FNR-Generating Activity: Fred

The prior purification protocol developed for Fred from camphor-grown *P. putida* NCIMB 10007 [15] was used to prepare and subsequently characterise highly purified preparations of the enzyme. Equivalent kinetic studies to those undertaken with the Frps deploying NADH or NADPH as the electron donor, FMN or FAD as the electron mediator and cytochrome c as the terminal acceptor under anaerobic conditions confirmed that Fred could only effectively transfer reducing power from NADH to FMN (*K_m_* FMN = 4.2 µM; *k_cat_* FMN = 294 s^−1^; *K_m_* NADH = 28 µM). Double reciprocal plots of the kinetic data generated by Fred as a function of NADH concentration in the presence of various fixed concentrations of FMN (Supplementary Figure S4a) demonstrated that Fred, like Frp1 and Frp2 and ActVB, exhibited kinetics indicating that the enzyme generated FNR by a sequential reaction mechanism.

As with the Frps, when assayed under aerobic conditions, Fred exhibited a marked progressive inhibition with added FMN concentrations higher than a threshold concentration (12.5–15 μM: Supplementary Figure S4b) due to the confirmed abiotic production of hydrogen peroxide from the resultant FNR.

#### 3.2.3. Inducible High MW FRs; PdR

Sufficient quantities of highly purified PdR to facilitate kinetic characterisation were prepared from camphor-grown *P. putida* NCIMB 10007 using the protocol previously reported by Willetts and Kelly [15]. Additionally, putidaredoxin (Pdx) was recovered as a by-product of the same protocol by deploying the method of Gunsalus and Wagner [29]. Equivalent kinetic studies to those undertaken both with Fred and the Frps under anaerobic conditions deploying NADH or NADPH as the electron donor, and either DCPIP or Pdx plus cytochrome c as the terminal acceptor, confirmed that PdR can only transfer reducing power from NADH as no detectible activity was recorded with NADPH. The recorded *K_m_* and *k_cat_* values for NADH-dependent reduction of Pdx plus cytochrome c (5.9 µM; 102 s^−1^) and DCPIP (7.9 µM; 82 s^−1^) correspond with this enzyme being a flavoprotein able to accept reducing power exclusively from NADH and thereby participate in the direct sequential intermolecular transfer of reducing power from NADH to Pdx. In this respect it differed from the other assayed FNR-generating enzymes in *P. putida* NCIMB 10007 that were able to generate freely diffusible FNR, and left unexplained the apparent ability of PdR to support DKCMO-dependent biooxidations.

**Figure 4:**
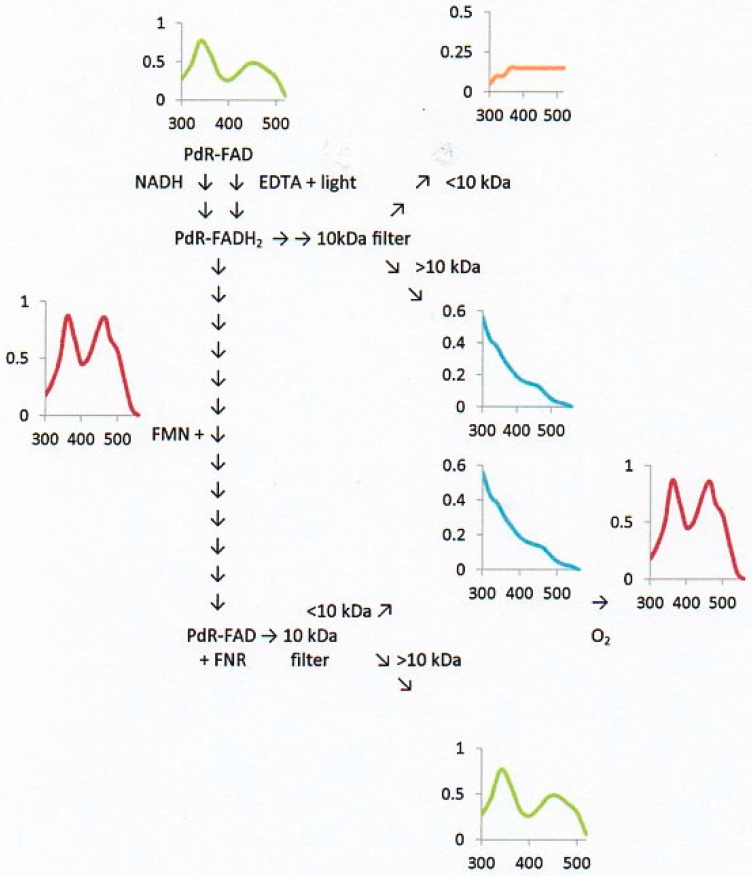
Initial transfer of reducing power from NADH to the bound flavin in putidaredoxin reductase (PdR) to form PdR-FADH_2_, and subsequent transfer from PdR-FADH_2_ to unbound FMN to form FNR. An equivalent redox transfer yielded freely diffusible FNR from PdR-FADH_2_ generated by prior photoreduction.

However, it was possible to demonstrate conclusively by characteristic changes in the relevant absorption spectra assayed at pH 7.6 under anaerobic conditions that FADH_2_, the bound low redox potential flavin coenzyme (midpoint E°′ = −0.28 V at pH 7.6; [40]) generated within PdR by NADH-dependent reduction was able to pass reducing power to unbound FMN added subsequently as a cofactor to the reaction mixture (midpoint E°′ = −0.20 V at pH 7.6; [40]). This transfer of reducing power from the bound lower to the unbound higher redoxpotential flavin, thereby generating FNR which remained freely diffusible in the reaction mixture, was evidenced both by the extent, and most significantly the location, of the relevant changes in the absorption spectra of the two flavins (Figure 4). On introducing oxygen to the <10 kDa filtrate of the final spent reaction mixture, FMN was regenerated and the resultant hydrogen peroxide formed confirmed using a pyrogallol and peroxidase-based test kit. An identical pattern of both the extent and distribution of absorbance changes occurred when photochemically reduced PdR [33] was substituted for NADH-reduced PdR (Figure 4), thereby eliminating the possibility of some uncharacterised form of direct transfer of reducing power from NADH to FMN.

### 3.3. Relevant Kinetic Data for the FNR-Dependent Isoenzymic DKCMO Activities in Camphor—Grown P. putida NCIMB 10007

The protocol previously developed by Willetts and Kelly [15] was scaled up to generate sufficient quantities of highly purified preparations of the isoenzymic DKCMOs to enable appropriate kinetic studies to be undertaken. Aspects of the selectivity of substrate binding were determined by exploiting a linked enzyme assay containing a purified sample of a DKCMO, an equivalent titre of either Fred or PdR or a mixture of Frp1 + 2 as the FR, the ketone substrate to be tested, and a linked in situ FNR-generating system consisting of FMN and NADH plus sodium formate and formate dehydrogenase. Ketone biooxidation and resultant lactone production were monitored aerobically at timed intervals for 360 min. The data obtained confirmed that both 2,5- and 3,6-DKCMOs were inactive with either enantiomer of fenchone, but were extremely selective both with respect to the corresponding diketocamphane enantiomers previously recovered from spent growth media, and the equivalent commercially available camphor antipodes (Table 1). For both isoenzymes, the corresponding chiral diketone proved to be the better substrate. Most significantly, these outcomes considerably extended the scope of DKCMOs to accept FNR generated by PdR as a source of the reducing power necessary to promote O_2_-dependendent lactonisations.

Because of the extended times taken for lactonisation reactions to go to completion, conventional Michaelis-Menton kinetic studies of the DKCMOs were considered likely to be unreliable. In order to obtain relevant data for the binding of the oxidised and reduced forms of the flavin cosubstrate, the methods previously reported for ActVA, the FNR-dependent monooxygenase component of the TCMO involved in actinorhodin biosynthesis in *Streptomyces coelicolor* [39] were used. Thus for FMN, the dissociation constant (*K_d_*) for binding for each DKCMO preparation was calculated from the fractional absorbance changes at 440 nm when assayed aerobically. Significantly, the relatively high values (2,5-DKCMO = 108.6 +/− 4.2 µM; 3,6-DKCMO = 125.3 +/− 5.1 µM : averages from 10 × replicates) suggested that FMN was bound only very weakly by either DKCMO isoenzyme.

However, as FNR does not exhibit marked absorbance bands, the equivalent *K_d_* values for FNR could not be obtained by the same spectrophometric titration method. Rather advantage was taken of the well-established ability of FNR to quench the fluorescence of tryptophan-containing apoproteins [34]: In this respect it is relevant that the 2,5- and 3,6-DKCMO isoenzymes contain nine and five tryptophan residues respectively per monomeric subunit. Assays were performed anaerobically using FNR stock solutions prepared by quantitative photoreduction in the presence of EDTA [33]. In contrast to the results obtained with FMN, the particularly low *K_d_* values obtained for FNR binding by both 2,5- and 3,6-DKCMO (2,5-DKCMO = 0.21 +/− 0.02 µM; 3,6-DKCMO = 0.24 +/− 0.02 µM: Averages from 10 × replicates) confirmed that for both isoenzymes, FNR was bound approximately 500-fold more tightly than FMN, with implications for the interaction of FNR within the respective active sites.

## 4. Discussion

As precedented by the pioneering studies of Jacob and Monod [41], the recorded pattern of diauxic growth of *P. putida* NCIMB 10007 on the composite succinate-camphor medium is a clear indication that this bacterium has developed mechanisms to regulate enzyme synthesis in response to changing conditions of demand. Crucial to this programme of adaptation to growth at the expense of (*rac*)-camphor will be the appearance in cells of the bacterium of significantly enhanced titres of the inducible enzymes of the convergent camphor degradation pathway thereby enabling metabolites derived from camphor to enter the TCA cycle. Two such key adaptive enzymes are 2,5-DKCMO and 3,6-DKCMO, the CAM plasmid-coded isoenzymic TCMOs that serve to delineate respectively the (+)- and (−)-camphor branches of the pathway. To function effectively, these FNR-dependent monooxygenases must be able to source freely diffusible reduced flavin cofactor from one or more concurrent and compatible FNR-generating enzymes. Such FRs can either be camphor-induced or constitutive. The outcomes of the linked enzyme biotransformation studies undertaken with purified preparations of the isoenzymic DKCMOs (Table 1) confirm that compatible FNR-generating activities for both TCMOs are the chromosome-coded enzymes Fred, Frp1 and Frp2, and the CAM plasmid-coded flavoprotein PdR, the redox intermediate subunit of heterotrimeric camphor *exo*-hydroxylase, the initiating enzyme of the (*rac*)-camphor degradation pathway. However, which if any of these TCMO-FR combinations functions effectively at any given time during camphor-dependent growth by *P. putida* NCIMB 10007 requires a more comprehensive analysis of the available data.

The initial phase of growth on the composite medium occurs exclusively at the expense of succinate, the more readily assimilatable carbon source, with no detectible induction of the isoenzymic DKCMOs, and therefore no directly related requirement for one or more of the compatible FNR-generating activities. A notable feature of this phase of growth is the presence of Frp1 and Frp2, two bi-functional enzymes each able to serve effectively either as a ferric reductase or a flavin reductase that can generate freely diffusible FNR from NADH by a sequential reaction mechanism. While no specific role for these two enzymes as FRs during succinate-dependent growth has been identified in the absence of the camphor-induced isoenzymic DCKMOs, the fact that both Frps remain equally active throughout the hiatus of diauxie and subsequent camphor-dependent growth suggests that these enzymes are constitutively expressed in *P. putida* NCIMB 10007, as are the equivalent chromosome-coded enzymes in *P. putida* KT2440 [16,17]. However, a primary role as ferric reductases, thereby ensuring the availability of intracellular ferrous ions, a necessity to ensure various FeII-dependent activities essential for successful aerobic growth [35], is entirely consistent with the constitutive nature of these bi-functional chromosome-coded enzymes.

Post-diauxic camphor-dependent growth results in a striking difference in the time-courses for the induction of the assayed CAM plasmid- and chromosome-coded FNR-related enzymes. The isoenzymic FNR-dependent DKCMOs (*camE_25-1_*, *camE_25-2_*, and *camE_36_* ) and FNR-generating PdR (*camA*) exhibit very similar parabolic-like patterns of induced activity throughout the various phases of camphor-dependent growth, as does heterotrimeric camphor *exo*-hydroxylase (*camA, camB , camC*) for which PdR serves as the NADH-dependent redox intermediate. However, chromosome-coded FR Fred [5] only begins to appear in cells that have progressed beyond the mid log-phase of camphor-dependent growth, which questions the functional significance of Fred as a supplier of FNR to the isoenzymic DKCMOs, at least in the early phases of camphor-dependent growth (vide supra).

The coincident time courses recorded for the induced titres of CAM plasmid-coded camphor *exo*-hydroxylase (*camA*, *camB*, *camC*), PdR (*camA*), and both isoenzymic DKCMOs (*camE_25-1_*, *camE_25-2_*, *camE_36_*) throughout all the phases of camphor-dependent growth by *P. putida* NCIMB 10007 is consistent with a comprehensive pattern of coordinate transcriptional control. In this respect it has already been established that *camA*, *camB*, and *camC*, along with the *camD* gene that codes for *exo*-hydroxycamphor dehydrogenase, constitute a single operon on the CAM plasmid subject to coordinate transcriptional control by the repressor protein product of the concatinated *camR* gene [42]. Significantly, a recent study [5] confirmed that the three relevant genes coding for 2,5-DKCMO and 3,6-DKCMO, plus the *camS* and *camV* genes that both code for transcriptional regulator proteins are also clustered close to the characterised *camRDCAB* operon. The fact that an almost identical pattern of inducible enzyme profiles is recorded when *P. putida* NCIMB 10007 is inoculated into a minimal medium containing succinate and 2-oxo-Δ^3^-4,5,5-trimethylcyclopentenylacetic acid (OTE) suggests that the specificity of the relevant genetic controls is sufficiently relaxed to include elements of product feedback induction. Relaxed inducer specificity allied to product feedback induction have been proposed previously to explain the induction of various hydroxycamphor dehydrogenases in *P. putida* NCIMB 10007 by OTE [7,8]. In strict contrast, the induction profile of Fred, a chromosome-coded FNR-generating enzyme, suggests that this FR not subject to any of these same elements of coordinate regulatory control in *P. putida* NCIMB 10007.

**Figure 5:**
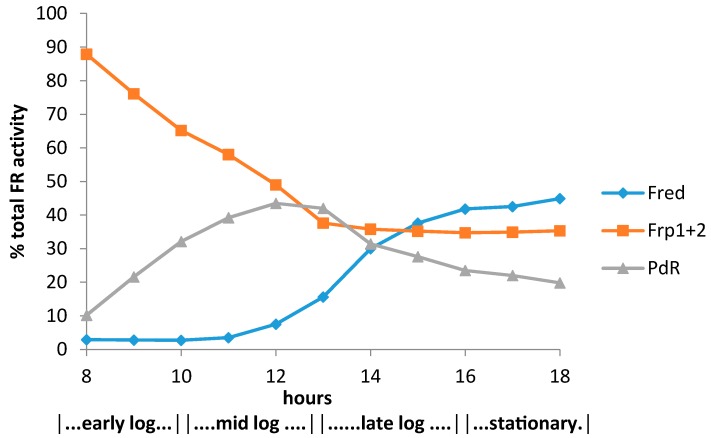
Relative contribution of the different assayed FNR-generating enzymes to the total flavin reductase activity titre throughout the various phases of camphor-dependent growth of *P. putida* NCIMB 10007.

The constitutive nature of the chromosome-coded Frps, and the significantly different time courses for the induction of plasmid-coded PdR and chromosome-coded Fred will clearly influence the likelihood of which, if any, of these activities can service the FNR dependency of the plasmid-coded DKCMOs at any given time throughout camphor-dependent growth by *P. putida* NCIMB 10007 in the composite medium. From the onset of camphor degradation to the transition from the mid log- to the late log-phase of camphor-dependent growth at around 12–13 h, the combined constitutive Frp1 + 2 activity is clearly a major FNR-generating activity (Figure 5). However, the combined Frp activity becomes progressively less predominant as the titre of plasmid-coded PdR increases until it reaches a maximum of approximately 45% of the total FR activity of cells as they enter into late log phase of growth. Most significantly, the absence of any significant titre of Fred eliminates this enzyme as a practical contender at these stages of camphor-dependent growth. Conversely, the profile of the PdR titre very closely matches that of both DKCMO isoenzymes (Figure 2), probably as a result of coordinate induction of the plasmid-coded genes by camphor (vide infra). Assuming that the relevant *K_m_* values for NADH of the various candidate activities are a reflection of their respective binding affinities [39], these data suggest that when assayed in isolation, the order of effectiveness for generating FNR during early log-phase camphor-dependent growth is PdR(5.9/7.9 µM) > Frp1(23 µM) > Frp2(36 µM), but conversely, based on the corresponding *k_cat_* values for the isolated enzymes, that order reverses to Frp2(358 s^−1^) > Frp1(310 s^−1^) > PdR(102/82 s^−1^). In all cases these data were generated under non-optimised conditions.

The late log and stationary phases of camphor-dependent growth are marked by some significant changes with respect to both the monitored FNR-generating and FNR-dependent activitities. Most interestingly, the late log phase of growth is characterised by the first significant appearance of chromosome-coded Fred, followed by a progressive rise of the relevant titre to thereby become the predominant FNR-generating activity, a trend which continues on into stationary phase (Figure 5). The comparative kinetic data generated under non-optimised conditions suggests that the relative order of effectiveness for generating FNR throughout post mid log phase camphor-dependent growth is PdR(5.9/7.9 µM) > Frp1(23 µM) > Fred (28 µM) > Frp2(36 µM) based on the relevant *K_m_* values for NADH, but reverses to Frp2(358 s^−1^) > Frp1(310 s^−1^ > Fred (294 s^−1^) > PdR (102/82 s^−1^) based on the relevant *K_cat_* values. However, the discontinuity in the timing of the induction of Fred relative to the DKCMOs (Figure 2) calls into question the functional relevance of this FR for these particular FNR-dependent enzymes. The time profile for the induction of Fred is more consistent with that of an enzyme involved in secondary rather than primary metabolism [38]. In that respect it is significant that species of *P. putida* are known to produce a wide range of indigenous secondary metabolites [43], including various polyketides [44], for which FR-linked TCMOs are known to play important roles as “tailoring enzymes” [24,25,26].

Although they are characterised by significantly different structural features (relevant MWs, and monomeric vs. dimeric) Fred and the two Frps, which are all chromosome-coded enzymes, do share some important common features when functioning as FRs. Firstly, all three enzymes operate sequential reaction mechanisms to generate freely diffusible FNR. Secondly, when assayed under aerobic conditions, they all exhibit significant progressive inhibition in the presence of FMN above a critical threshold concentration because the resultant level of the accumulating FNR product is sufficient to trigger autooxidation of the flavin, thereby generating hydrogen peroxide which then detrimentally effects further FR activity. Frp2 is also noteworthy in being the only FNR-generating system able to generate FNR from NADPH as well as NADH, although it is not possible to establish from these limited kinetic data obtained (*K_m_* NADH = 36 µM; NADPH = 92 µM) whether any of the resultant freely diffusible NADPH-derived FNR is used as a source of reducing power by the DKCMOs under physiological conditions. In this respect it is significant that all the previous studies that have concluded that the DKCMOs from camphor-grown *P. putida* NCIMB 10007 are ultimately NADH-dependent monooxygenases have been undertaken exclusively in conjunction with the 36 kDa NADH-dependent enzyme sourced from late log phase cells. However, unlike Frp2, this enzyme which was previous termed NADH oxidase [45] or NADH dehydrogenase [46] but has recently been confirmed to be the flavin reductase Fred [5], is totally inactive with NADPH.

That a purified preparation of PdR from camphor-grown *P. putida* NCIMB 10007 can generate freely diffusible FNR is the first clear demonstration that this CAM plasmid-coded activity can act as a FR in addition to its established role as a flavoprotein redox intermediate for camphor *exo*-hydroxylase [47]. The outcomes of the coupled PdR-DKCMO biotransformations with both isoenzymic DKCMOs demonstrate the ability of the plasmid-coded flavoprotein to act as an effective FR for promoting key lactonization steps in the pathways for both (+)- and (−)-camphor degradation. These data also explain why less highly purified preparations of the DKCMOs, known to contain unresolved PdR [13], were able to promote various nucleophilic [13,14,48] and electrophilic [27] biooxidations without supplementation by an established FNR-generating enzyme. The significance of this newly characterised activity for PdR is that it increases the biochemical autonomy of the CAM plasmid as it is now possible to assign plasmid-coded proteins acting as enzymes and/or redox intermediates for every activity necessary to metabolise both camphor antipodes to the level of HTP-CoA (Figure 1). It still remains to be established how the available pool of PdR-FADH_2_ could be effectively divided by Pdx to support two very different outcomes.

As the sole FNR-dependent activities studied, the plasmid-coded DKCMOs yield some significant outcomes. Each isoenzyme while exhibiting absolute stereoselectivity can lactonise both the DKC and camphor antipode within the relevant series. Interestingly, Ornston’s proposal [49] for relaxed forms of regulatory control for some degradative pathways in *Pseudomonas* spp. includes broad substrate specificity of relevant enzymes as a significant contributory factor that complements the type of comprehensive pattern of coordinate transcriptional control established in (*rac*)-camphor-grown *P. putida* NCIMB 10007 (*vide infra*). Within each enantiomeric series, both corresponding activities biotransform the relevant bornadione at a faster rate than the bornanone, which probably reflects the relative binding characteristics of the di- and monoketone substrates in the active sites of the DKCMOs, which in turn suggests that initial hydroxylation and subsequent dehydrogenation prior to lactonisation may provide the kinetically predominant route in vivo for the degradation of both antipodes of camphor by *P. putida* NCIMB 10007 (Figure 1). Because of a combination of the extended times for lactonisation reactions to go to completion and the extreme susceptibility of unbound FNR to autooxidation, obtaining a relevant comprehensive kinetic data set for the flavin dependency of the DKCMOs is problematical. However, by taking advantage of characteristic changes to the absorption and fluorescence spectra resulting from flavin binding to the tryptophan-containing DKCMOs, it is possible to obtain relevant *K_d_* values for both FMN and FNR. Both DKCMO isoenzymes exhibited a very large difference (> 500-fold) in the respective *K_d_* values for FNR and FMN, resulting in each case from a very low value for FNR compared to a notably high value for FMN. Equivalent but smaller comparative differences (~200-fold) have been reported for other flavin-dependent TCMOs [9,50]. In each case, these very high differences most probably reflect the significant difference in the three-dimensional shape of the oxidised and reduced forms of the isoalloxazine tricyclic ring, the key functional feature common to all flavins, and how this relates to the topography of the active sites of the relevant enzymes. The conjugated tricyclic ring in FMN is planar, whereas in FNR it is bowed through the N5–N10 axis into the so-called “butterfly” conformation [51,52].This difference has been used as a key factor in solving the crystal structure of very many flavin-dependent TCMOs. Although similarity between them is generally low, the overall pattern of structural folding of the resolved enzymes is well preserved [53]. Above all, the feature of H-bonding interactions between FNR N5-H and a hydroxyl group of a serine or threonine residue in the active site, which is crucial for subsequent C4a-hydroperoxyflavin formation and stabilisation, is conserved in all established structures [54].

These data are particularly relevant to the recently proposed three-dimensional crystallographic structural model of 3,6-DKCMO from *P. putida* NCIMB 10007 [55]. The model predicts that there is no significant difference in the hydrogen bonding patterns of FMN and FNR in the active site of the enzyme, which does not correspond with the >500-fold difference in the actual *K_d_* values for the two forms of the flavin recorded with this enzyme. This fundamental inconsistency with the experimentally determined data may help to explain why the proposed 3,6-DKCMO model fails to demonstrate the crucial H-bonding interactions between FNR N5-N and a hydroxyl-containing residue in the proposed active site which are an established key functional feature of every other TCMO characterised at this level [54]. These discrepancies may in part reflect that Isupov et al’s model is based on relatively low-grade data, with resolution only being achieved by Molecular Replacement based on a synthetic α_2_ version of the luciferase from *Vibrio harveyi* [56]. However, this α/β heterodimeric light-emitting enzyme is a different type of TCMO: most significantly, the β-subunit, although catalytically inactive itself, dynamically interacts with the α-subunit thereby increasing the stability and quantum efficiency of the latter by 5+ orders of magnitude [57,58], a feature that the synthetic α_2_ dimer does not take into account. Further, Isupov et al. modelled FNR binding in the active site of oxygen-dependent 3,6-DKCMO on the precedent of Adh, again a different type of TCMO, that catalyses F_420_-dependent dehydrogenation with no dependency on either FNR or oxygen [59]. Consequently, the model is likely to be a poor basis for gaining insight into the molecular mode of action of 3,6-DKCMO from camphor-grown *P. putida* NCIMB 10007.

So, while the current study has characterised a number of interesting features of the interplay between the CAM plasmid- and chromosome-coded enzymes of early part of the convergent pathway of camphor degradation in *P. putida* NCIMB 10007, including elements of the relevant kinetic and regulatory controls, further studies including relevant genetic approaches may help to generate a more comprehensive understanding of the whole pathway to the points of entry of the carbon atoms of the bornane ring into the TCA cycle.

## Figures and Tables

**Table 1:** Biotransformations of mono- and diketone chiral substrates by various diketocamphane monooxygenase-flavin reductase combinations.

Enzyme Combination	(+)Camphor % Oxidation (%ee Lactone Product) 180 m 360 m	(−)Camphor % Oxidation (%ee Lactone Product) 180 m 360 m	2,5-DKC % Oxidation (%ee Lactone Product) 180 m 360 m	3,6-DKC % Oxidation (%ee Lactone Product) 180 m 360 m	(+)Fenchone % Oxidation (%ee Lactone Product) 180 m 360 m	(−)Fenchone % Oxidation (%ee Lactone Product) 180 m 360 m
25DKCMO +	58 90	0 0	66 100	0 0	0 0	0 0
Fred	(96) (98)		(na) (na)			
25DKCMO +	62 95	0 0	69 100	0 0	0 0	0 0
Frp1 & 2	(94) (98)		(na) (na)			
25DKCMO +	48 86	0 0	54 90	0 0	0 0	0 0
PdR	(92) (97)		(na) (na)			
36DKCMO +	0 0	60 92	0 0	76 100	0 0	0 0
Fred		(94) (96)		(na) (na)		
36DKCMO +	0 0	58 95	0 0	76 100	0 0	0 0
Frp1 & 2		(96) (98)		(na) (na)		
36DKCMO +	0 0	49 86	0 0	62 93	0 0	0 0
PdR		(93) (97)		(na) (na)		

## Supplementary Materials

The following is available online at www.mdpi.com/2076-2607/4/4/38/s1: Figure S1: changes in the optical density (A_500_ nm), succinate (mM), (*rac*)-camphor (mM), and the specific activity of camphor *exo*-hydroxylase (*camA, camB, camC*) and its component flavoprotein redox intermediate PdR (*camA*) during diauxic growth of *P. putida* NCIMB 10007 on a camphor plus succinate-based defined medium; Figure S2: changes in the optical density (A_500_ nm) and the specific activity of key enzymes of camphor degradation during diauxic growth of *P. putida* NCIMB 10007 on a succinate plus 2-oxo-∆^3^ -4,5,5-trimethylcyclopentenylacetic acid (OTE)-based defined medium; Figure S3: (a)Frp1 initial velocity as a function of NADH concentration in the presence of 3.0, 6.0, 9.0 and 12.0 μM FMN; (b) Frp2 initial velocity as a function of NADH concentration in the presence of 4.0, 8.0, 12.0 and 16.0 μM FMN; (c) Effect of [FMN] on the activity of Frp1 and Frp2; Figure S4: (a) Fred initial velocity as a function of NADH concentration in the presence of 4.0, 6.0, 8.0, and 12 μM FMN; (b) Effect of [FMN] on the activity of Fred.
