# Supplementary material for: Flavin-Dependent Redox Transfers by the Two-Component Diketocamphane Monooxygenases of Camphor-Grown Pseudomonas putida NCIMB 10007
Source: Microorganisms. 2016 Oct 13;4(4):38. doi: 10.3390/microorganisms4040038 (PMC5192521; doi:10.3390/microorganisms4040038)
Supplement: Supplementary file 1 [file microorganisms-04-00038-s001.pdf]

Supplementary Materials: Flavin-Dependent Redox Transfers by the Two-Component Diketocamphane Monooxygenases of Camphor-Grown *Pseudomonas putida* NCIMB 10007. *Microorganisms* 2016, 4, 38

Andrew Willetts and David Kelly

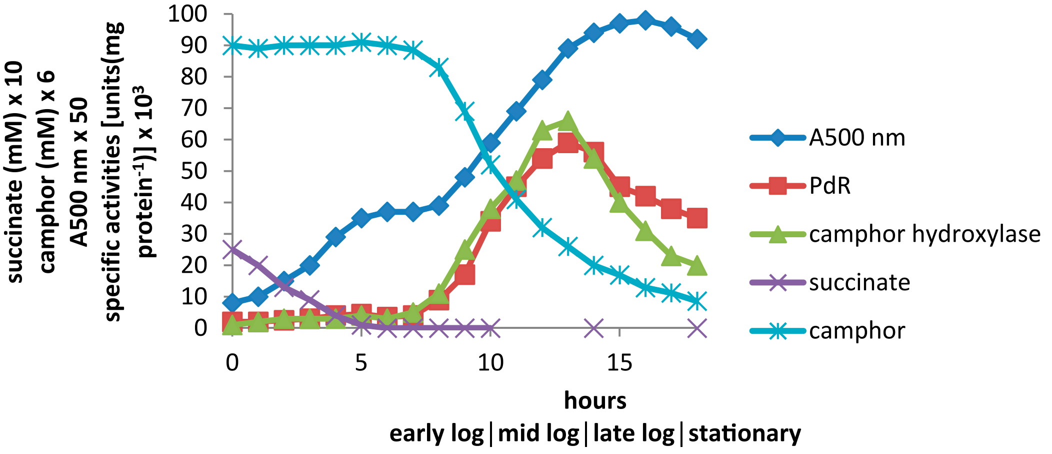

Figure S1. Changes in the optical density (A_{500} nm), succinate (mM), (*rac*)-camphor (mM), and the specific activity of camphor *exo*-hydroxylase (*camA, camB, camC*) and its component flavoprotein redox intermediate PdR (*camA*) during diauxic growth. of *P putida* NCIMB 10007 on a camphor plus succinate-based defined medium.

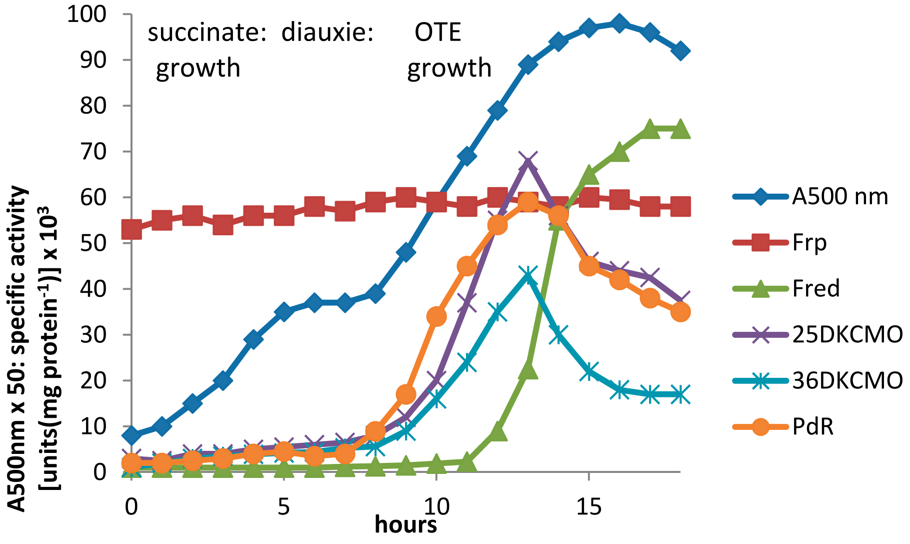

Figure S2. Changes in the optical density (A_{500} nm) and the specific activity of key enzymes of camphor degradation during diauxic growth of *P putida* NCIMB 10007 on a succinate plus 2-oxo-Δ^3 -4,5,5-trimethylcyclo- pentenylacetic acid (OTE)-based defined medium.

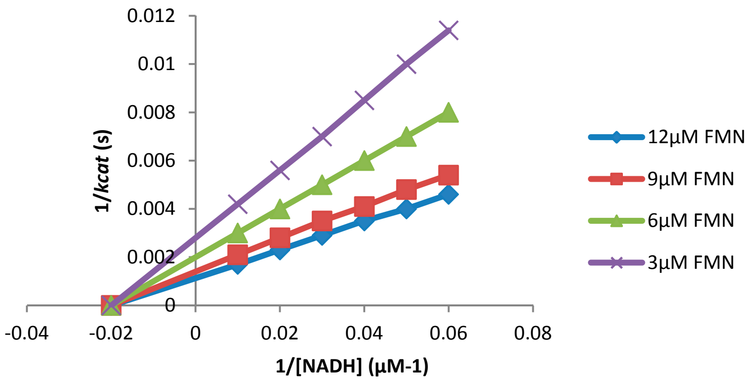

(a)

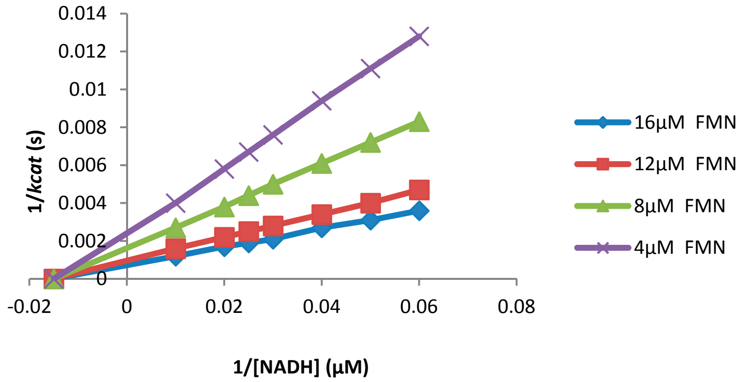

(b)

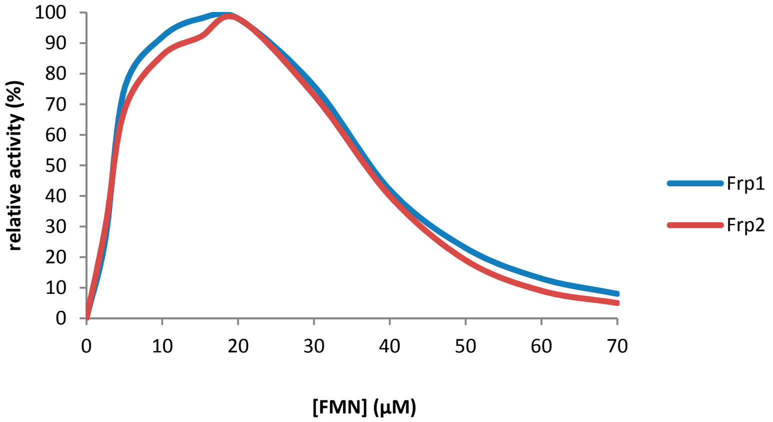

(c)

Figure S3. (**a**)Frp1 initial velocity as a function of NADH concentration in the presence of 3.0, 6.0, 9.0 and 12.0 µM FMN; (**b**) Frp2 initial velocity as a function of NADH concentration in the presence of 4.0, 8.0, 12.0 and 16.0 µM FMN; (**c**) Effect of [FMN] on the activity of Frp1 and Frp2.

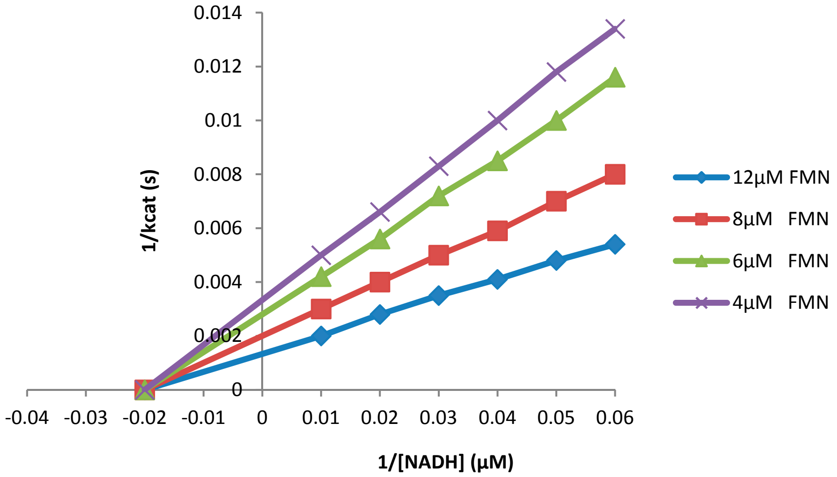

(a)

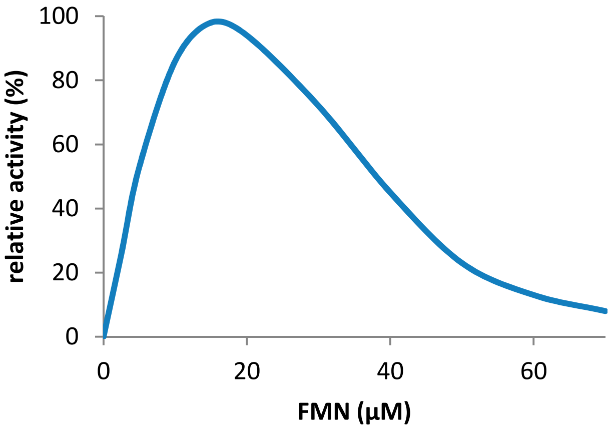

(b)

Figure S4. (**a**) Fred initial velocity as a function of NADH concentration in the presence of 4.0, 6.0, 8.0, and 12 µM FMN; (**b**) Effect of (FMN) on the activity of Fred.

© 2016 by the authors; licensee MDPI, Basel, Switzerland. This article is an open access article distributed under the terms and conditions of the Creative Commons by Attribution (CC-BY) license (http://creativecommons.org/licenses/by/4.0/).
